# Laparoscopic jejunostomy for enteral nutrition in gastric cancer patients: A report of two cases: A case report

**DOI:** 10.1016/j.ijscr.2022.107388

**Published:** 2022-07-06

**Authors:** Hiroshi Tsuchiya, Itaru Yasufuku, Naoki Okumura, Nobuhisa Matsuhashi, Takao Takahashi

**Affiliations:** Department of Gastroenterological and Pediatric Surgery, Gifu University Graduate School of Medicine, 1-1 Yanagido, Gifu-City, Gifu-Prefecture 501-1194, Japan

**Keywords:** Laparoscopic jejunostomy, Gastric cancer

## Abstract

**Introduction and importance:**

Jejunostomy is often indicated for patients with oral intake difficulties and unresectable gastric cancer, patients at risk of postoperative complications, and patients who require nutritional management after gastrectomy. In this report, we discuss the cases with laparoscopic jejunostomy in our department.

**Case presentation:**

Case 1: An upper gastrointestinal endoscopy performed for close examination in a 60-year-old male revealed upper gastric cancer with extensive invasion and lower esophageal stenosis. He had difficulty with esophageal transit and, consequently, underwent a laparoscopic jejunostomy and staging laparoscopy.

Case 2: Upper gastrointestinal endoscopy in a 62-year-old male revealed type 3 tumor in the gastric antrum. He had a history of chronic obstructive pulmonary disease requiring home oxygen therapy, pulmonary hypertension, and heart failure, and was at a high perioperative risk. Consequently, both laparoscopic distal gastrectomy and laparoscopic jejunostomy were performed.

**Clinical discussion:**

Enteral nutrition has many advantages over venous nutrition, including maintenance of immunity and intestinal mucosa, avoidance of bacterial translocation, and decreased risk of catheter infection. Although there are a few reports of cases with laparoscopic jejunostomy, it is expected that the technique will become more widespread and safe in the future.

**Conclusion:**

Laparoscopic jejunostomy is considered a useful, minimally invasive, and safe technique.

## Highlights

• We report two cases wherein laparoscopic jejunostomy was useful in gastric cancer.
• Laparoscopic jejunostomy is useful, minimally invasive, and safe.
• This technique may become more widespread and safer in the future.

## 1 Introduction

In recent years, minimally invasive surgery, typified by laparoscopic surgery, has been increasingly indicated for the treatment of advanced gastrointestinal cancer. Jejunostomy is performed to manage enteral nutrition in patients who have oral intake difficulty due to upper gastrointestinal tract obstruction or who are at increased risk due to surgical, therapeutical, or iatrogenic complications. Noting the rarity of reported cases, we describe two cases in which laparoscopic jejunostomy was useful in gastric cancer patients. This work has been reported in line with the SCARE criteria [23].

## 2 Presentation of case

### 2.1 Case 1: a 60-year-old male

Chief complaint: lightheadedness.

Current medical history: The patient was referred to our department with a diagnosis of advanced gastric cancer with extensive esophageal invasion.

**Fig. 1:**
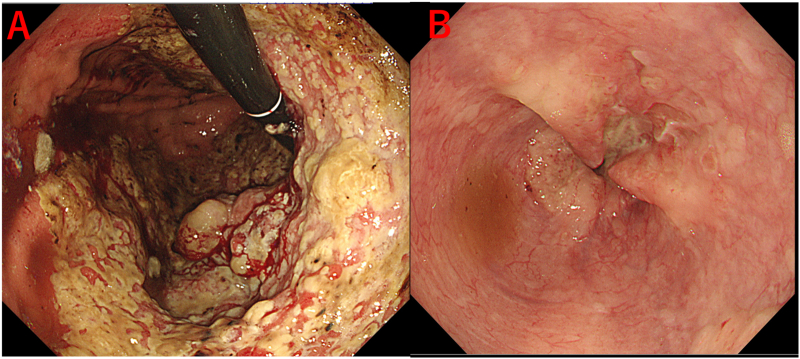
Upper gastrointestinal endoscopic findings. A-The circumferential stenotic typ4 tumor was found in the upper gastric body. B-The tumor extensively invades the esophagus with stenosis.

Upper gastrointestinal endoscopic findings: A type 4 tumor with circumferential stenosis in the gastric cardia was observed, which was barely passable through a narrow scope (Fig. 1A, B). Biopsy results showed poorly differentiated adenocarcinoma.

Contrast-enhanced computed tomography (CT) scan: There was no obvious evidence of distant metastasis.

Blood tests: albumin concentration: 3.0 g/dl, hemoglobin (Hb) level: 9.2 g/dl, and ChE: 134 U/l.

Tumor markers: CEA: 227.7 ng/ml and CA: 19–9 3.8 U/ml.

Clinical diagnosis: Gastric cancer, UME, Circ, type 4, por, cT4aN0M0, and cStage IIB (Gastric Cancer Treatment Code, 15th edition). A staging laparoscopy was scheduled to evaluate local invasion and peritoneal dissemination. If radical resection was difficult, we planned a simultaneous laparoscopic jejunostomy to avoid malnutrition caused by transit disturbance.

**Fig. 2:**
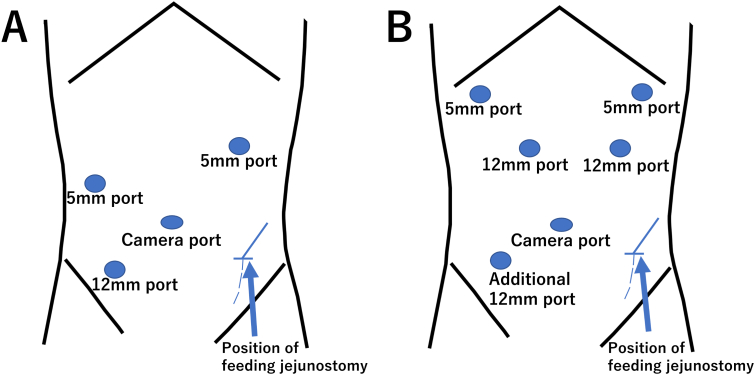
Port placement. A-The operator used the two ports on the right side. B-We added a port in the right lower abdomen after distal gastrectomy.

**Fig. 3:**
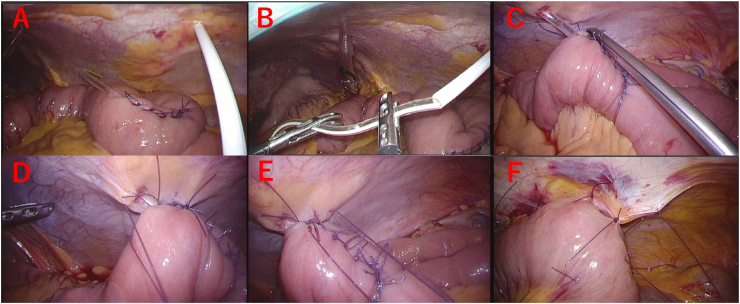
Laparoscopic jejunostomy technique. A, B-We used a puncture needle to puncture the abdominal wall and guide the tube out of the body cavity. C, D, E-The jejunum was fixed to the abdominal wall with three sutures surrounding the jejunostomy puncture site. F-Approximately 3 cm jejunum was fixed to the abdominal wall on the oral and anal sides, respectively.

Surgical findings: A 12-mm camera port was placed in the umbilicus, a 5-mm port (surgeon's left hand) and 12-mm port (surgeon's right hand) were placed at the right side of the abdomen, and a 5-mm port (assistant) was placed at the left side of the abdomen (Fig. 2A). Intra-abdominal observation revealed numerous peritoneal seeding nodules in the right and left subdiaphragmatic spaces, Douglas fossa, and small intestine mesentery. There was no indication for radical resection, and the decision to create a jejunostomy was made. We inserted a jejunostomy catheter into the jejunum approximately 40 cm from the ligament of Treitz. We extended the umbilical skin incision by 4 cm in the cephalocaudal direction, guided the jejunum out of the peritoneal cavity, inserted a 9 Fr enterostomy catheter outside the peritoneal cavity, fixed it to the intestinal wall using the Witzel technique, returned the jejunum and catheter into the peritoneal cavity, and resumed insufflation. The catheter was guided out of the abdominal wall through the outer tube using a catheter introducer puncture needle to guide it to the left side of the abdomen, as caudally as possible (Fig. 3A, B). The catheter was secured with a 3–0 synthetic absorbable thread with three stitches around the jejunal entry point of the enterocutaneous catheter (Fig. 3C, D, E), each approximately 3 cm long from the insertion site to the mouth and anus (Fig. 3F).

Postoperative course: Enteral nutrition using a jejunostomy was started the day after surgery. The patient was discharged on the 10th postoperative day without complications, along with instructions on how to manage the jejunostomy at home, and was promptly transferred to chemotherapy as an outpatient. Palliative chemotherapy was continued; however, the patient died 9 months after surgery. Jejunostomy was used, without complications until the end of the patient's life.

### 2.2 Case 2: a 62-year-old male

Chief complaint: lightheadedness.

**Fig. 4:**
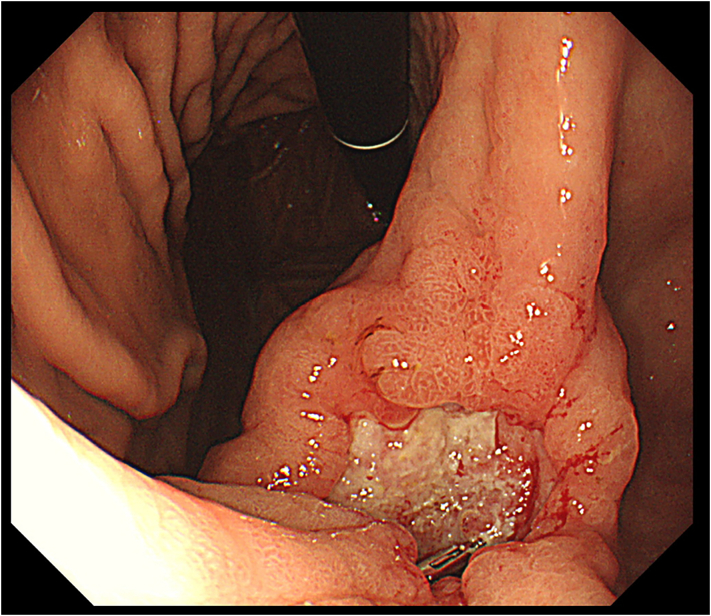
Upper gastrointestinal endoscopic findings. The type3 tumor was found in the lesser curvature of the gastric angle.

Current medical history: The patient had chronic obstructive pulmonary disease (COPD), pulmonary hypertension, and heart failure, and was receiving nasal 3 l/min home oxygen therapy while visiting the Department of Cardiology. A blood test was performed after he felt lightheaded, which revealed prominent anemia (Hb level: 6.4 g/dl). An upper gastrointestinal endoscopy was performed for close examination, which revealed a type 3 tumor on the anterior wall of the gastric antrum (Fig. 4). He was referred to our department for surgery.

History: COPD (on home oxygen therapy), pulmonary hypertension, heart failure, and deep vein thrombosis (on ELIQUIS®, apixaban).

Upper gastrointestinal endoscopy revealed a type 3 tumor 40 mm in size on the anterior wall of the gastric antrum. Biopsy results showed a moderately-differentiated adenocarcinoma.

Contrast-enhanced CT scan: There was wall thickening with contrast effect in the lesser curvature of the gastric horn, but no obvious lymph node metastasis or distant metastasis was observed.

Tumor markers: CEA: 1.6 ng/ml and CA: 19–9 8.3 U/ml.

Preoperative diagnosis: Gastric cancer, M, Ant, type 3, tub2, cT2N0M0, and cStage I (Gastric Cancer Treatment Code, 15th Edition). The patient was considered to be at a high risk for perioperative complications due to respiratory and cardiovascular comorbidities; thus, we decided to perform a simultaneous jejunostomy for postoperative nutritional management.

Surgical findings: A 12-mm camera port was placed in the umbilicus, 12-mm ports were placed at the right and left sides of the abdomen, and 5-mm ports were placed at the right and left sacral ribs (Fig. 2B), as in a conventional laparoscopic distal gastrectomy. The tumor was located on the anterior wall of the gastric antrum, with a trailing serous membrane, and was determined to be T3 (invading the subserosa, SS) in depth. The patient underwent distal gastrectomy, D2 lymph node dissection, and Roux-en-Y reconstruction with anterior colonic route. A 4-cm-long umbilical skin incision was extended in the cephalocaudal direction for the removal of the intraoperative resected stomach and anastomosis of the Y leg. The jejunum was dissected outside the peritoneal cavity approximately 20 cm from the Treitz ligament with a linear stapler, and a lateral anastomosis was performed 45 cm from the raised jejunal distal end. A jejunostomy catheter was inserted into the jejunum 20 cm anorectally from the Y leg, and the catheter was fixed to the intestinal wall with a Witzel suture. After returning to intracorporeal manipulation and completing the reconstruction, the catheter was guided out of the peritoneal cavity from the left lower abdomen and fixed to the abdominal wall by the same procedure as in Case 1. A 12-mm port was added to the right lower abdomen (Fig. 2B).

Postoperative course: Enteral nutrition using jejunostomy was started on the second postoperative day. The patient was discharged home on the 15th postoperative day after instruction on jejunostomy catheter management. Four weeks after surgery and as soon as oral intake stabilized, the enterocutaneous fistula was removed on an outpatient basis. The patient has been free of jejunostomy-related complications for 12 months.

## 3 Discussion

Enteral nutrition reportedly has many advantages over intravenous nutrition, including immune and intestinal mucosa support, avoidance of bacterial translocation, and decreased risk of venous catheter infection [1], [2], [3], [4], [5]. Jejunostomy is an alternative to the oral administration of enteral nutrients. Systemic chemotherapy is the first-line treatment for unresectable advanced gastric cancer [6]. Laparoscopic jejunostomy was first reported by O'Regan et al. in 1990 [7], and since then, the results of laparoscopic jejunostomy have been reported mainly overseas [8], [9].

In the primary endpoint of overall survival, the randomized, controlled, phase III REGATTA trial (JCOG 0705/KGCA1) of in situ resection before chemotherapy in patients with unresectable advanced gastric cancer did not demonstrate superiority over the standard of care (chemotherapy alone) [10]. This trial showed no significant difference in overall survival between the two chemotherapeutic regimens. Although the study excluded patients with impaired gastrointestinal transit, it was noted that primary chemotherapy was administered less frequently in patients undergoing epigastric resection, especially in those with tumors located in the epigastric region [10]. In such patients, gastric jejunal anastomosis is difficult, and there is a need for a less invasive and early postoperative feeding method than epigastric total resection, which can be tube-feeding. Laparoscopic enterostomy may be an effective method of nutritional administration that is less invasive than surgical total gastrectomy and allows tube feeding in the early postoperative period. In recent years, regimens that can be administered intravenously without oral anticancer drugs, such as FOLFOX therapy, have been indicated in the primary treatment of unresectable advanced gastric cancer [6]. Furthermore, more than 50 % of patients with unresectable advanced gastric cancer who have difficulty with oral intake due to tumor-induced transit disturbance have reported that oral intake is possible after FOLFOX therapy [11], [12]. For patients with advanced unresectable gastric cancer with dyspepsia, a laparoscopic jejunostomy may be an option for treatment. In a retrospective study, it was reported that there were fewer postoperative complications with laparoscopic jejunostomy than with the open approach [13]. Especially in advanced gastric cancer, the results of the JLSSG0901 study showed that laparoscopic surgery is not inferior to open surgery; thus, the percentage of laparoscopic gastric cancer surgery is expected to increase in the future. However, Yung et al. reported that the complication rate of laparoscopic jejunostomy was 4 % within 30 days of surgery and 8.7 % post-surgery [11]. In particular, complications due to infection of the puncture site were relatively frequent, ranging from 0.7 % to 12.5 % [8], [9], [14], [15], [16], and were thought to be because the Witzel suture was not sutured intracorporeally or adequately, or that it was not performed at all. Postoperative bowel obstruction was a frequent complication of the open approach jejunostomy in esophageal cancer patients, ranging from 4.5 % to 11.5 % [17], [18], [19]. The most common cause of jejunostomy-related obstruction is torsion of the small bowel around the abdominal fixation site [20]. Suture fixation of the small bowel in its natural position and over a length of approximately 4 cm at the lateral abdomen was reported as effective. In some cases, when the small intestine is fixed to the upper abdomen, the distal portion overcomes the abdominally fixated one, resulting in flexion and obstruction of the small intestine. Intestinal obstruction related to fixation on the abdominal wall is also an important complication of enterocutaneous fistula creation.

Since laparoscopic Witzel suture is rather complicated, we guided the small intestine out of the body cavity through the umbilical port, inserting a jejunostomy catheter under direct vision, and we performed a Witzel to ensure fixation. These procedures can be performed with only a slight lengthening of the umbilical opening and are considered to be useful. We have not had any cases of postoperative intestinal obstruction to date.

The number of laparoscopic procedures for various diseases and organs has been increasing in Japan in recent years [22]. Additionally, the results of the JLSSG0901 study reported at the 94th Annual Meeting of the Japanese Gastric Cancer Association demonstrated non-inferiority of laparoscopic distal gastrectomy to open approaches in advanced gastric cancer in terms of the 5-year recurrence-free survival rate. Another advantage of laparoscopic jejunostomy is that it can be performed simultaneously with staging laparoscopy. Although there are a few reports of laparoscopic jejunostomy, it is expected that the technique will become more widespread and safer in the future.

## 4 Conclusion

Laparoscopic jejunostomy is considered a useful technique that is minimally invasive and safe.

## Consent

Written informed consent was obtained from the patients for publication of this report and accompanying images. A copy of the written consent is available for review by the Editor-in-Chief of this journal on request.

## Declaration of competing interest

The authors declare that they have no competing interests.
